# Influence of lymph node removal on the prognosis of high malignancy potential gastric gastrointestinal stromal tumors: Insights from population-based study

**DOI:** 10.1371/journal.pone.0314504

**Published:** 2024-12-05

**Authors:** Zhenguo Qiao, Zhi Zhang, Junjie Chen, Ping Yin, Xin Ling, Weihai Chen, Lingxia Yang

**Affiliations:** 1 Department of Gastroenterology, Suzhou Ninth People’s Hospital, Suzhou Ninth Hospital Affiliated to Soochow University, Suzhou, China; 2 Department of General Surgery, Suzhou Ninth People’s Hospital, Suzhou Ninth Hospital Affiliated to Soochow University, Suzhou, China; 3 Department of Traditional Chinese Medicine, Wujiang Fifth People’s Hospital, Suzhou, China; 4 Department of Cardiology, Suzhou Ninth People’s Hospital, Suzhou Ninth Hospital Affiliated to Soochow University, Suzhou, China; Universitá Sapienza di Roma, ITALY

## Abstract

High malignancy potential gastric gastrointestinal stromal tumors (HMP-gGISTs) generally require surgical resection. However, the necessity of lymph node removal (LR) for patients with such tumors remains unclear. Therefore, we conducted a population-based study to analyze the impact of LR on the long-term prognosis of patients with HMP-gGISTs. Patients with HMP-gGISTs were gathered from the Surveillance, Epidemiology, and End Results (SEER) database. Propensity score matching (PSM) was utilized to address potential selection bias. Overall survival (OS) and cancer-specific survival (CSS) were evaluated using Kaplan-Meier analyses and multivariate Cox proportional hazards models. A total of 840 patients with HMP-gGISTs were included in the study, with 317 undergoing LR and 523 not undergoing LR. The prognosis for OS (*P* = 0.026) and CSS (*P* < 0.001) in the LR group was worse compared to the No-LR group. After PSM, 634 patients were matched for comparison. The results showed that the OS (*P* = 0.028) and CSS (*P* = 0.006) in the LR group remained poorer than those in the No-LR group. Subgroup analysis further indicated that patients who did not undergo LR had a better prognosis. Our findings suggest that LR may not improve the prognosis of patients with HMP-gGISTs, implying that LR may not be necessary for these patients.

## Introduction

Gastrointestinal stromal tumors (GISTs) are the most common mesenchymal neoplasms originating from the gastrointestinal tract, accounting for 1%-3% of gastrointestinal tumors [1,2]. The majority of these tumors are found in the stomach (40–51%), followed by the small intestine (20–40%), with other locations including the colon/rectum and the retroperitoneum [3–5]. Despite the improved prognosis for GIST patients with the introduction of imatinib [6,7], surgical intervention remains the primary treatment for GIST [8,9]. Due to the localized growth pattern of GISTs and their rare occurrence of lymph node metastasis, routine lymph node removal is not recommended. Güller et al. [10] reported that lymph node metastasis was present in 5.1% of GIST patients in the Surveillance, Epidemiology, and End Results (SEER) database. Other studies with small sample sizes have shown varying rates of lymph node metastasis in GIST patients, ranging from 8.8% to 20.7% [11,12]. The presence of lymph node metastasis is associated with poorer overall survival in patients with metastatic GISTs [13].

In addition to being more likely to occur in succinate dehydrogenase (SDH) deficiencies [14,15] and at a younger age [16,17], lymph node metastasis in GIST patients may also be associated with the level of malignancy. Several risk stratification systems have been proposed to assess the recurrence risk after the complete resection of primary GIST, including the modified National Institutes of Health (NIH) classification system [18], American Forces Institute of Pathology (AFIP) criteria [19], and National Comprehensive Cancer Network (NCCN) Guideline biological behavior predictor system [20]. Among these systems, the modified NIH criteria are the most commonly used for evaluating the potential malignant risk of GISTs. They categorize them into four groups (very low, low, intermediate, and high risk) based on tumor size, mitotic count, tumor site, and tumor rupture [18]. For patients with low malignancy potential gastric gastrointestinal stromal tumors (LMP-gGISTs), endoscopic treatment offers a favorable long-term prognosis due to the extremely low risk of lymph node metastasis [21]. On the contrary, for patients with high malignancy potential gastric gastrointestinal stromal tumors (HMP-gGISTs), there is an increased risk of tumor metastasis and postoperative recurrence [22]. However, it is currently uncertain whether lymph node resection during surgery would affect the long-term prognosis of HMP-gist patients. Therefore, utilizing the SEER database, this study conducted a comparative analysis of the long-term prognosis between HMP-gist patients with lymph node resection and those without, with the aim of providing guidance for the surgical strategy in such cases.

## Methods

### Study population

**Fig 1:**
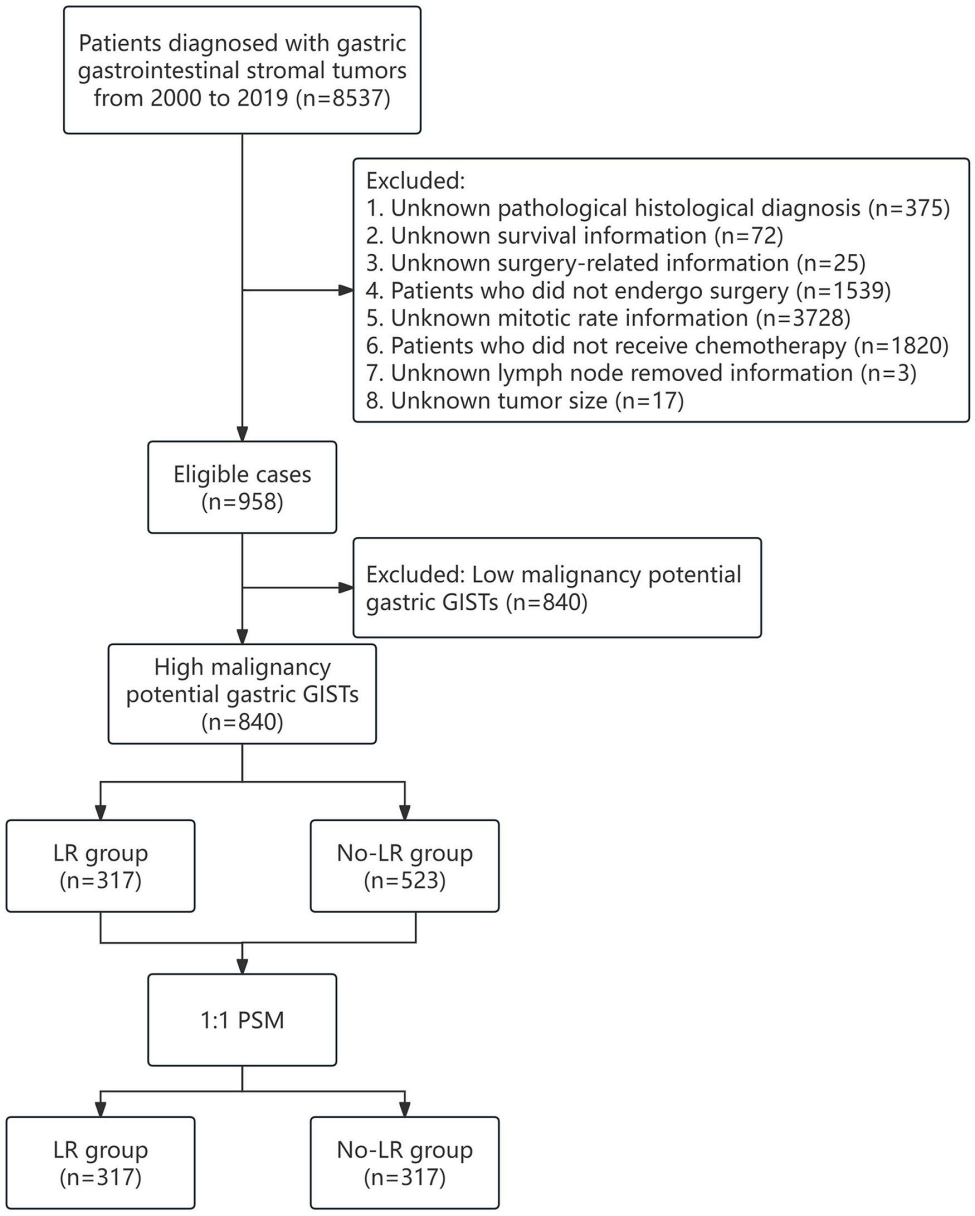
Flow chart of the study.

Patients diagnosed with GIST between 2000 and 2019 were selected from the recently updated SEER database, which covers approximately 28% of the US population and provides comprehensive data on various cancers. The SEER program’s official website offers a wealth of resources, including user manuals, tutorials, and guides to aid in the effective understanding and utilization of the data. The database’s codebook serves as a detailed manual, providing information on the encoding and interpretation of each variable in the database. GISTs were identified based on the International Classification of Diseases for Oncology, Third Edition (ICD-O-3), using the unique histological subtype code 8936. The exclusion criteria consisted of: (1) unknown pathological histological diagnosis; (2) unknown survival information; (3) unknown surgery-related information; (4) patients who did not undergo surgery; (5) unknown mitotic rate information; (6) patients who did not receive chemotherapy; (7) unknown information on lymph nodes removed; (8) unknown tumor size. Fig 1 illustrates a flowchart outlining the protocol followed in the study. The study extracted several covariates, such as age, race, sex, tumor grade, marital status, tumor size, mitotic rate, cancer-specific mortality, overall survival status, and duration of follow-up. Participants were categorized into two age groups: those 60 years old or younger (referred to as the young group) and those older than 60 years (referred to as the elderly group). With regards to race, individuals were classified into white, black, or other races (including American Indian, Alaska Native, and Asian/Pacific Islander). Tumor size was divided into four groups: <2.0, 2.1–5.0, 5.1–10.0, and >10.0. The study used the variable "CS site-specific factor 6" to assess the mitotic rate. Surgery-related information was determined using RX Summ-Surg Prim Site (1998+) codes, and whether LR was performed was determined using RX Summ—Scope Reg LN Sur. The analysis calculated overall survival (OS) and cancer-specific survival (CSS) from the GIST diagnosis until death, death due to cancer, or the last follow-up date. According to the modified NIH criteria (see S1 Table), the very low/low-risk group is defined as having low malignancy potential, while the intermediate/high-risk group is defined as having high malignancy potential. Since the SEER database contains only anonymized, publicly accessible data, the research did not directly involve human subjects, eliminating the need for Institutional Review Board (IRB) approval and informed consent.

### Multiple imputation

The research encountered instances of incomplete data in various variables, including race (1.0% of cases), tumor grade (44.6%), and marital status (5.5%). To address this issue, a polytomous regression model was utilized, incorporating the multiple imputation (MI) technique in R software (version 4.1.0). This method was implemented to improve the statistical analysis of the study.

### Statistical analysis

Categorical variables were presented as frequencies and percentages, and group comparisons were conducted using the chi-square test. Continuous variables with non-normal distributions were described using medians and interquartile ranges (IQR), and the Mann-Whitney U test was used for comparative analyses. One-to-one PSM was employed to balance the LR and No-LR groups. The propensity model included age, race, sex, tumor grade, marital status, tumor size, and mitotic rate, with a caliper width set at 0.01. OS and CSS were calculated using the Kaplan-Meier method, and group comparisons were made using the log-rank test. Multivariate Cox proportional hazard models were used to assess hazard ratios (HRs) and 95% confidence intervals (CIs). All statistical analyses were performed using R software (version 4.0). A significance threshold of *P* < 0.05 was set to determine significant differences between groups.

## Results

### Patient characteristics

A total of 840 patients diagnosed with HMP-gGISTs were included in the study, with 317 (37.7%) undergoing LR and 523 (62.3%) not undergoing LR. Prior to PSM, a significant difference (P < 0.05) was observed between the LR and No-LR groups in terms of patient sex and tumor size. The median (IQR) follow-up period for the LR and No-LR groups was 64.0 (43.0–94.5) months and 67.0 (46.0–95.0) months, respectively (*P* = 0.277). Following PSM, the analysis included 317 matched pairs of patients, with no significant differences observed in any baseline characteristics between the two groups (Table 1). Detailed demographic and clinical data for the patients are presented in Table 1, while S2 Table provides a comprehensive comparison of the demographic and clinical features of the two groups before matching. In S3 Table, out of the 317 patients who underwent LR, a total of 308 patients had examination of regional nodes, with only 23 patients (7.3%) testing positive for regional nodes.

**Table 1:** Comparison of the demographic and clinical characteristics between LR and No-LR group in patients with gastric gastrointestinal stromal tumors before and after PSM.

Variables	Before PSM				After PSM		
	Total (n = 840)	LR group (n = 317)	No-LR group (n = 523)	*P*-value	LR group (n = 317)	No-LR group (n = 317)	*P*-value
Sex				0.003			0.263
Male	429 (51.1%)	183 (57.7%)	246 (47.0%)		183 (57.7%)	169 (53.3%)	
Female	411 (48.9%)	134 (42.3%)	277 (53.0%)		134 (42.3%)	148 (46.7%)	
Age				0.380			0.382
≤60	368 (43.8%)	145 (45.7%)	223 (42.6%)		145 (45.7%)	156 (49.2%)	
>60	472 (56.2%)	172 (54.3%)	300 (57.4%)		172 (54.3%)	161 (50.8%)	
Race				0.120			0.625
White	495 (58.9%)	174 (54.9%)	321 (61.4%)		174 (54.9%)	174 (54.9%)	
Black	210 (25.0%)	91 (28.7%)	119 (22.8%)		91 (28.7%)	83 (26.2%)	
Others	135 (16.1%)	52 (16.4%)	83 (15.9%)		52 (16.4%)	60 (18.9%)	
Grade				0.079			0.998
Well	179 (21.3%)	57 (18.0%)	122 (23.3%)		57 (18.0%)	57 (18.0%)	
Moderately	344 (41.0%)	124 (39.1%)	220 (42.1%)		124 (39.1%)	126 (39.7%)	
Poorly	111 (13.2%)	49 (15.5%)	62 (11.9%)		49 (15.5%)	49 (15.5%)	
Undifferentiated	206 (24.5%)	87 (27.4%)	119 (22.8%)		87 (27.4%)	85 (26.8%)	
Size				0.005			0.895
<2.0	6 (0.7%)	2 (0.6%)	4 (0.8%)		2 (0.6%)	1 (0.3%)	
2.1–5.0	91 (10.8%)	22 (6.9%)	69 (13.2%)		22 (6.9%)	20 (6.3%)	
5.1–10.0	375 (44.6%)	135 (42.6%)	240 (45.9%)		135 (42.6%)	142 (44.8%)	
>10.0	368 (43.8%)	158 (49.8%)	210 (40.2%)		158 (49.8%)	154 (48.6%)	
Marital status				0.981			0.741
Married	541 (64.4%)	204 (64.4%)	337 (64.4%)		204 (64.4%)	200 (63.1%)	
Unmarried	299 (35.6%)	113 (35.6%)	186 (35.6%)		113 (35.6%)	117 (36.9%)	
Mitotic rate				0.348			0.811
≤5	452 (53.8%)	164 (51.7%)	288 (55.1%)		164 (51.7%)	167 (52.7%)	
>5	388 (46.2%)	153 (48.3%)	235 (44.9%)		153 (48.3%)	150 (47.3%)	
Survival months, median (IQR)	66.0(45.0,94.8)	64.0(43.0,94.5)	67.0(46.0,95.0)	0.277	64.0(43.0,94.5)	68.0(47.5,92.0)	0.240

PSM: Propensity score matching; LR: Lymph node removed; Others: American Indian, Alaska Native, Asian/Pacifc Islander; HPF: High power field; IQR: Interquartile range.

### Comparison between the LR and No-LR groups on OS and CSS

**Fig 2:**
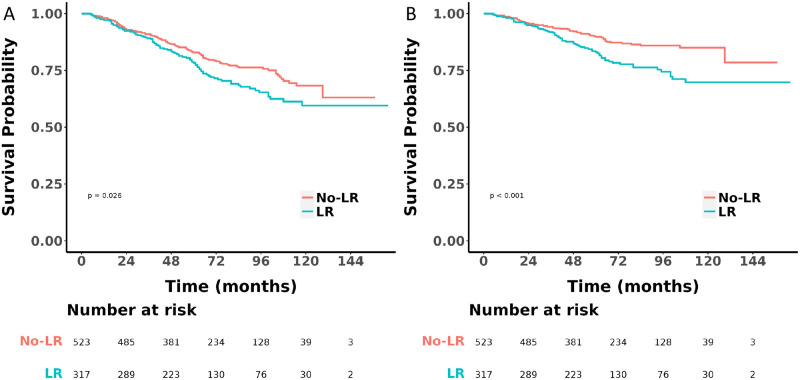
OS and CSS were compared before PSM A. OS; B CSS OS: Overall survival; CSS: Cancer-specific survival; PSM: Propensity score matching.

**Fig 3:**
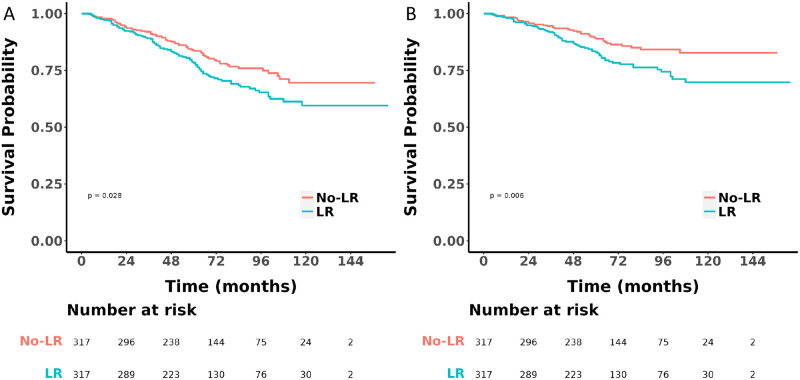
OS and CSS were compared after PSM A. OS; B CSS OS: Overall survival; CSS: Cancer-specific survival; PSM: Propensity score matching.

During the median follow-up period of 66.0 months (IQR 45.0 to 94.8 months), there were 91 deaths in the LR group, with 66 attributed to HMP-gGISTs, and 114 deaths in the No-LR group, with 61 attributed to HMP-gGISTs. Prior to PSM, Kaplan-Meier analysis and log-rank tests indicated that the OS (HR 1.37, 95% CI 1.04–1.80, *P* = 0.026) (Fig 2A) and CSS (HR 1.85, 95% CI 1.31–2.62, *P* < 0.001) (Fig 2B) were worse in the LR group compared to the No-LR group. After PSM, 634 patients (equally matched between the LR and No-LR groups) were compared. The OS (HR 1.42, 95% CI 1.04–1.80, *P* = 0.028) (Fig 3A) and CSS (HR 1.73, 95% CI 1.17–2.56, *P* = 0.006) (Fig 3B) of the LR group continued to be inferior to those of the No-LR group.

### Univariate and multivariate cox regression

Univariate Cox regression analysis indicated that not performing LR is beneficial for OS (HR 0.70, 95% CI 0.51–0.96, P = 0.028) and CSS (HR 0.58, 95% CI 0.39–0.86, P = 0.006) among patients with HMP-gGISTs. Additionally, multivariate Cox regression analysis also demonstrated that not performing LR can improve patients’ OS (HR 0.70, 95% CI 0.51–0.96, *P* = 0.025) and CSS (HR 0.55, 95% CI 0.37–0.82, *P* = 0.003) (Tables 2 and 3).

**Table 2:** Univariate and multivariate cox regression for analyzing the overall survival for patients with gastric gastrointestinal stromal tumors.

Variables	Univariate			Multivariate		
	HR	95% CI	*P*-value	HR	95% CI	*P*-value
Sex						
Male	1	Reference		-	-	-
Female	0.82	0.59–1.12	0.213	-	-	-
Age						
≤60	—	—		1	Reference	
>60	1.81	1.31–2.51	<0.001	1.95	1.40–2.72	<0.001
Race						
White	1	Reference		-	-	-
Black	1.40	1.00–1.96	0.051	-	-	-
Others	0.73	0.44–1.20	0.210	-	-	-
Grade						
Well	1	Reference		1	Reference	
Moderately	2.88	1.60–5.19	<0.001	3.27	1.81–5.92	<0.001
Poorly	2.63	1.36–5.07	0.004	2.53	1.30–4.91	0.006
Undifferentiated	2.38	1.28–4.43	0.006	2.48	1.33–4.63	0.004
Size						
<2.0	1	Reference		1	Reference	
2.1–5.0	0.11	0.02–0.62	0.012	0.18	0.03–1.06	0.054
5.1–10.0	0.31	0.08–1.28	0.106	0.28	0.07–1.16	0.079
>10.0	0.41	0.10–1.68	0.216	0.37	0.09–1.56	0.178
Marital status						
Married	1	Reference		1	Reference	
Unmarried	1.37	1.01–1.88	0.049	1.33	0.96–1.83	0.085
Mitotic rate						
≤5	1	Reference		-	-	-
>5	1.36	0.99–1.86	0.054	-	-	-
Lymph node removed						
Yes	1	Reference		1	Reference	
No	0.70	0.51–0.96	0.028	0.70	0.51–0.96	0.025

HPF: High power field; Others: American Indian, Alaska Native, Asian/Pacifc Islander; HR: Hazard ratios.

**Table 3:** Univariate and multivariate cox regression for analyzing the cancer-specific survival for patients with gastric gastrointestinal stromal tumors.

Variables	Univariate			Multivariate		
	HR	95% CI	*P*-value	HR	95% CI	*P*-value
Sex						
Male	1	Reference		-	-	-
Female	0.77	0.52–1.14	0.198	-	-	-
Age						
≤60	1	Reference		-	-	-
>60	1.13	0.77–1.65	0.543	-	-	-
Race						
White	1	Reference		-	-	-
Black	1.16	0.76–1.77	0.494	-	-	-
Others	0.69	0.38–1.26	0.228	-	-	-
Grade						
Well	1	Reference		1	Reference	
Moderately	5.14	2.05–12.88	<0.001	5.92	2.36–14.86	<0.001
Poorly	4.82	1.81–12.83	0.002	4.67	1.74–12.55	0.002
Undifferentiated	4.30	1.67–11.09	0.003	4.54	1.76–11.71	0.002
Size						
<2.0	1	Reference		1	Reference	
2.1–5.0	0.06	0.01–0.43	0.005	0.16	0.01–1.04	0.056
5.1–10.0	0.21	0.05–0.86	0.030	0.27	0.06–1.12	0.072
>10.0	0.30	0.07–1.22	0.092	0.38	0.09–1.58	0.186
Marital status						
Married	1	Reference		-	-	-
Unmarried	1.29	0.88–1.90	0.197	-	-	-
Mitotic rate						
≤5	1	Reference		-	-	-
>5	1.37	0.94–2.01	0.105	-	-	-
Lymph node removed						
Yes	1	Reference		1	Reference	
No	0.58	0.39–0.86	0.006	0.55	0.37–0.82	0.003

HPF: High power field; Others: American Indian, Alaska Native, Asian/Pacifc Islander; HR: Hazard ratios; bold values indicate *P* < 0.05.

### Subgroup analysis

**Fig 4:**
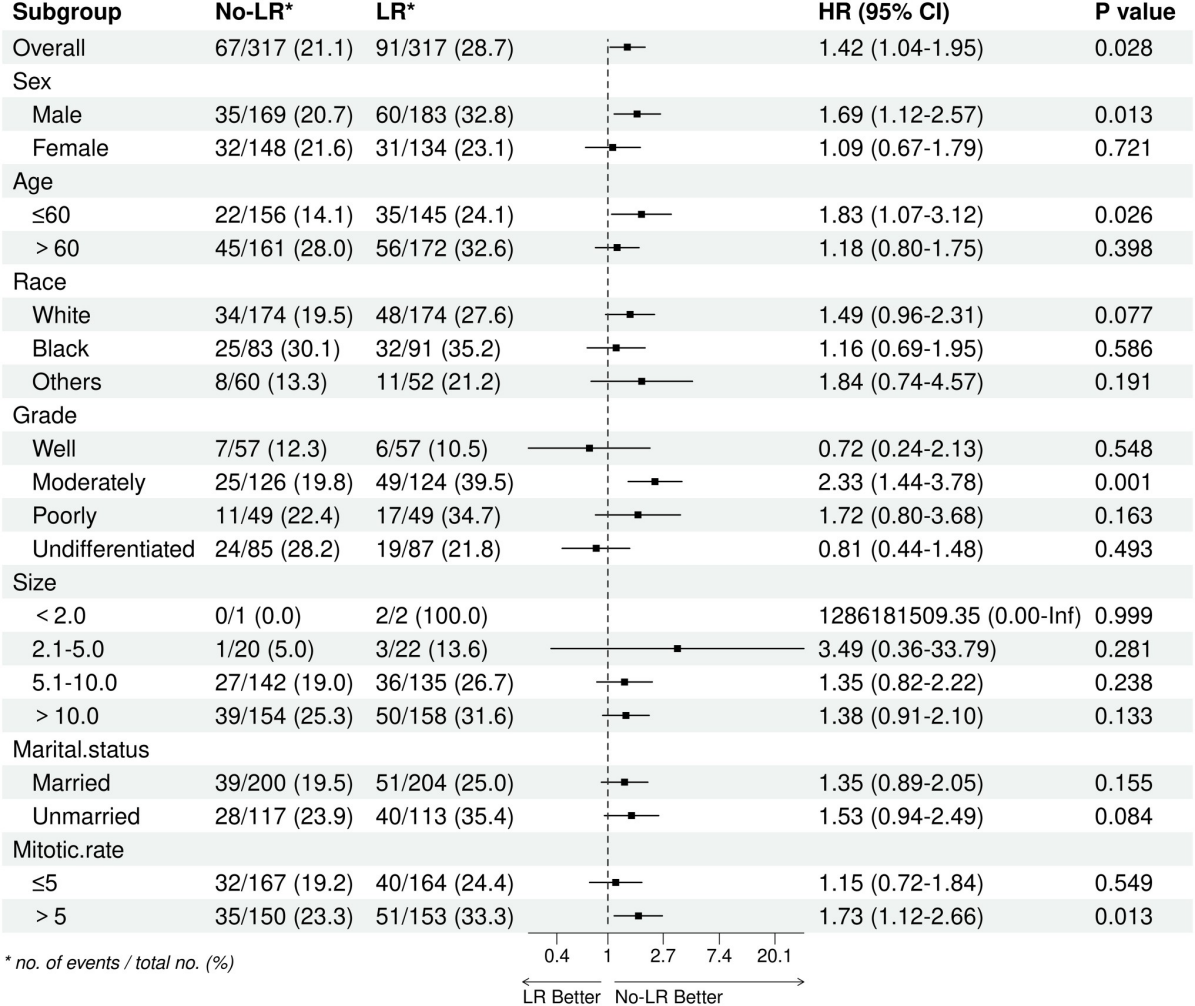
Subgroup analysis of overall survival between LR group and No-LR group after PSM. PSM: Propensity score matching; LR: Lymph node removed.

**Fig 5:**
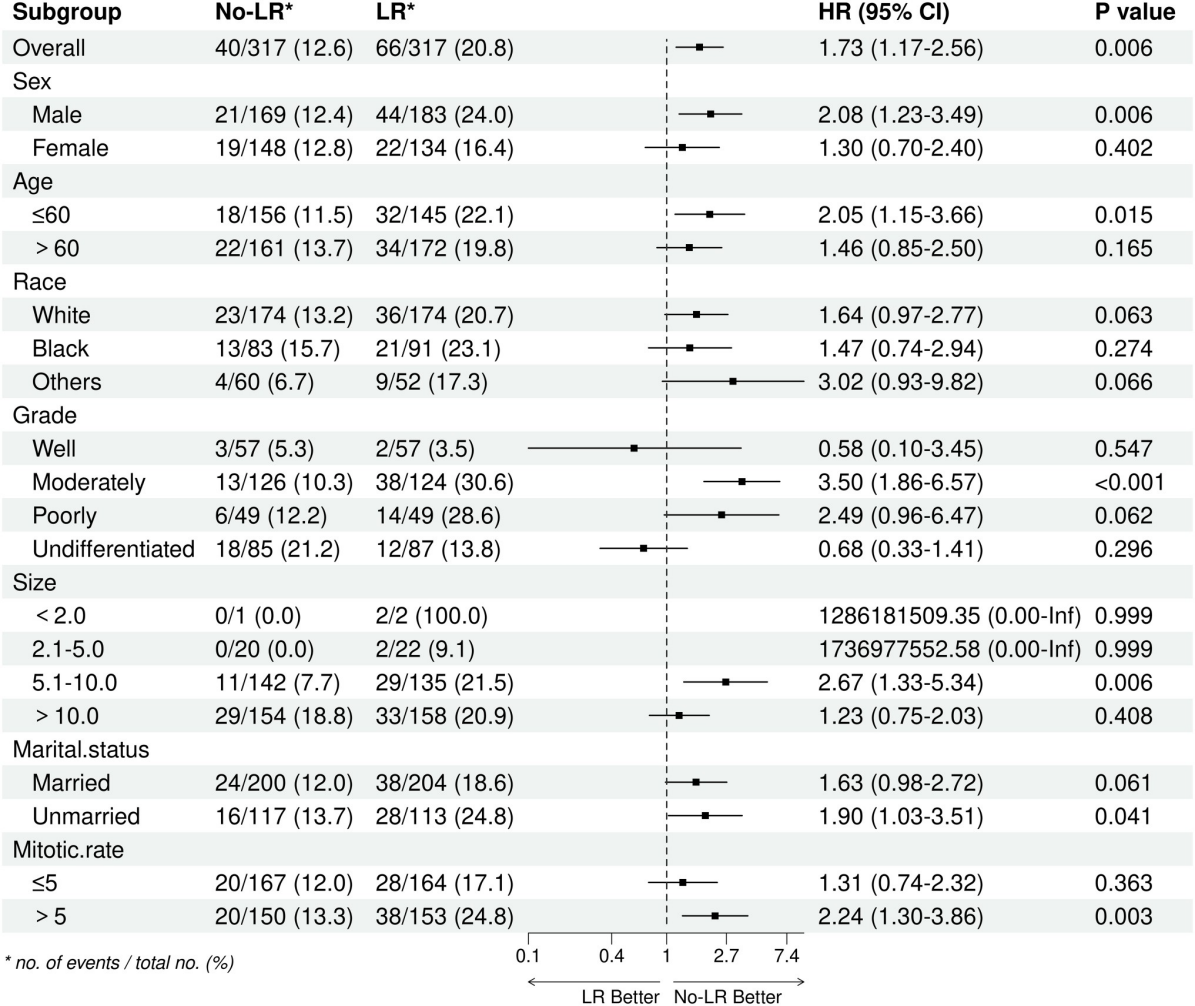
Subgroup analysis of cancer-specific survival between LR group and No-LR group after PSM. PSM: Propensity score matching; LR: Lymph node removed.

After performing PSM, most of the subgroup analyses showed a higher OS rate in patients who did not undergo LR, especially in males, those aged 60 years or younger, and those with a mitotic rate exceeding 5/50HPF (Fig 4). Additionally, the analyses consistently demonstrated improved CSS in the No-LR group compared to the LR group, with similar trends observed among the aforementioned subgroups (Fig 5).

## Discussion

Consistent with methodologies used in previous research [23–26], our study categorizes patients with intermediate/high-risk GISTs as HMP-gGISTs based on modified NIH criteria. Tumor size and mitotic rate are key factors in determining the malignant potential of gGISTs. Tumors measuring between 2.0 and 5.0 cm with a mitotic rate of over 5/50 HPF have a metastasis or tumor-related mortality rate of 16%, while those ranging from 2.0 to 10.0 cm with a mitotic rate below 5/50 HPF show a metastasis or tumor-related mortality rate below 4% [19,27]. Patients with intermediate and high-risk GISTs are at significantly higher risk of recurrence after surgical resection [22,28], indicating not only a greater risk of metastasis but also a worse prognosis for those with HMP-gGISTs [20]. Therefore, improving the prognosis for these patients is a key focus of current research. Some studies have suggested that minimally invasive treatments, such as endoscopic or laparoscopic approaches, may lead to a more favorable prognosis for individuals with LMP-gGISTs [21,29–32]. However, enhancing the prognosis for patients with HMP-gGISTs remains an important area of investigation. Surgical resection and adjuvant therapy with imatinib are commonly used approaches to managing HMP-gGISTs [33–36]. Therefore, in this study, we excluded cases that did not receive chemotherapy after surgery. Research by Joensuu et al. [35,37] has shown that postoperative imatinib treatment can improve the overall survival of GIST patients. However, the impact of different surgical approaches on the prognosis of HMP-gGISTs patients is still uncertain. Therefore, this study conducted a comparative analysis of the long-term prognosis between patients undergoing LR and those not undergoing LR.

Our study found that regardless of whether it was before or after PSM, the OS and CSS of the LR group were worse than those of the No-LR group (Figs 2 and 3). We believe that for patients with HMP-gGISTs, the poorer prognosis of the LR group compared to the No-LR group may be due to: (1) LR surgery is relatively complex, which may lead to more surgical complications such as bleeding, infection, anastomotic fistula, etc. These complications may prolong the patient’s recovery time, increase postoperative mortality, and thus affect prognosis. At the same time, S3 Table shows that only 23 patients (7.3%) were positive among the 308 patients who underwent regional nodes examined. (2) Lymph nodes are an important component of the immune system, and lymph node dissection may lead to a decrease in immune system function, weakening the body’s immune surveillance and clearance of tumor cells, thereby increasing the risk of recurrence [38,39].

Surgical resection typically focuses on the primary tumor without routine LR [20], resulting in limited research on LR for gastric stromal tumors. Tokunaga et al. [11] documented 57 cases of gGISTs undergoing surgery with LR, with only 5 cases (8.8%) showing lymph node metastasis. However, all 5 patients succumbed within 5 years post-surgery, despite 3 having undergone curative surgery. Corresponding with our study’s conclusion, Kubota et al. indicated that systematic LR might not be a feasible option, and the prognosis of GIST patients may not hinge on whether LR is performed [40]. Nonetheless, the aforementioned studies were confined to individual case reports and small sample sizes. To the best of our knowledge, our study represents the first population-based investigation comparing the long-term prognosis between the LR group and the No-LR group in HMP-gGISTs patients. Alongside the modified NIH criteria, as well as those of AFIP and NCCN, which stress the significance of tumor size and mitotic rate in GIST patient prognosis, our study unveiled that age over 60 and grade are also pivotal factors influencing prognosis for HMP-gGISTs patients.

Our study has several limitations. Firstly, it is a retrospective analysis conducted using the SEER database, which inherently leads to potential data omissions and biases. Nonetheless, we employed MI and PSM techniques to mitigate the effects of missing data and selection bias. Secondly, The SEER database does not contain data regarding postoperative complications, recurrence, margin status, chemotherapy regimen, and genetic mutations of KIT or PDGFRA, all of which may have an impact on the long-term prognosis of patients. Thirdly, information regarding tumor rupture status in GIST surgery patients is absent from the SEER database, and utilizing our own clinical data for subsequent analysis might allow for improved stratification of patients according to their malignant potential. Lastly, the duration of adjuvant treatment (imatinib) is unknown, which could impact survival, as there are studies supporting the use of 1, 2, 3, 5, or even 6 years of adjuvant therapy, and this could influence survival outcomes.

In summary, our study compared the long-term prognosis of HMP-gGISTs patients undergoing surgical treatment with or without LR using the SEER database. We found that LR may not improve the prognosis of patients with HMP-gGISTs. Therefore, we recommend that these patients do not require LR during surgery. However, further collection of clinical information is necessary to further evaluate the impact of LR on the prognosis of HMP-gGISTs patients.

## Supporting information

S1 Table Modified NIH classification system. (DOCX)

S2 Table Comparison of the demographic and clinical characteristics between LR and No-LR group in patients with gastric gastrointestinal stromal tumors before multiple imputation. (DOCX)

S3 Table Information related to the LR group. (DOCX)

S1 Data The data. (XLSX)
