# Supplementary material for: Influence of lymph node removal on the prognosis of high malignancy potential gastric gastrointestinal stromal tumors: Insights from population-based study
Source: PLoS One. 2024 Dec 5;19(12):e0314504. doi: 10.1371/journal.pone.0314504 (PMC11620419; doi:10.1371/journal.pone.0314504)
Supplement: S1 Table — (DOCX) [file pone.0314504.s001.docx]

**Supplementary Table 1.** Modified NIH classification system

| Risk category | Tumor size (cm) | Mitotic index (per 50 HPFs) | Primary tumor site |
| --- | --- | --- | --- |
| Very-low risk | <2.0 | ≤5 | Any |
| Low risk | 2.1-5.0 | ≤5 | Any |
| Intermediate risk | 2.1-5.0  <5.0  5.1-10.0 | >5  6-10  ≤5 | Gastric  Any  Gastric |
| High risk | Any  >10.0  Any  >5.0  2.1-5.0  5.1-10.0 | Any  Any  >10  >5  >5  ≤5 | Tumor rupture  Any  Any  Any  Nongastric  Nongastric |

NIH: National Institutes of Health; HPF: high power field
