# Supplementary material for: Influence of lymph node removal on the prognosis of high malignancy potential gastric gastrointestinal stromal tumors: Insights from population-based study
Source: PLoS One. 2024 Dec 5;19(12):e0314504. doi: 10.1371/journal.pone.0314504 (PMC11620419; doi:10.1371/journal.pone.0314504)
Supplement: S2 Table — (DOCX) [file pone.0314504.s002.docx]

**Supplementary Table 2.** Comparison of the demographic and clinical characteristics between LR and No-LR group in patients with gastric gastrointestinal stromal tumors before multiple imputation

| Variables | Total  (n=840) | LR group  (n=317) | No-LR group  (n=523) | *P*-value |
| --- | --- | --- | --- | --- |
| **Sex** |  |  |  | 0.003 |
| Male | 429 (51.1%) | 183 (57.7%) | 246 (47.0%) |  |
| Female | 411 (48.9%) | 134 (42.3%) | 277 (53.0%) |  |
| **Age** |  |  |  | 0.380 |
| ≤60 | 368 (43.8%) | 145 (45.7%) | 223 (42.6%) |  |
| ＞60 | 472 (56.2%) | 172 (54.3%) | 300 (57.4%) |  |
| **Race** |  |  |  | 0.107 |
| White | 489 (58.2%) | 173 (54.6%) | 316 (60.4%) |  |
| Black | 210 (25.0%) | 91 (28.7%) | 119 (22.8%) |  |
| Others | 133 (15.8%) | 52 (16.4%) | 81 (15.5%) |  |
| Unknown | 8 (1.0%) | 1 (0.3%) | 7 (1.3%) |  |
| **Grade** |  |  |  | 0.005 |
| Well | 113 (13.5%) | 29 (9.1%) | 84 (16.1%) |  |
| Moderately | 181 (21.5%) | 67 (21.1%) | 114 (21.8%) |  |
| Poorly | 64 (7.6%) | 29 (9.1%) | 35 (6.7%) |  |
| Undifferentiated | 107 (12.7%) | 53 (16.7%) | 54 (10.3%) |  |
| Unknown | 375 (44.6%) | 139 (43.8%) | 236 (45.1%) |  |
| **Size** |  |  |  | 0.005 |
| ＜2.0 | 6 (0.7%) | 2 (0.6%) | 4 (0.8%) |  |
| 2.1-5.0 | 91 (10.8%) | 22 (6.9%) | 69 (13.2%) |  |
| 5.1-10.0 | 375 (44.6%) | 135 (42.6%) | 240 (45.9%) |  |
| ＞10.0 | 368 (43.8%) | 158 (49.8%) | 210 (40.2%) |  |
| **Marital status** |  |  |  | 0.901 |
| Married | 501 (59.6%) | 191 (60.3%) | 310 (59.3%) |  |
| Unmarried | 293 (34.9%) | 110 (34.7%) | 183 (35.0%) |  |
| Unknown | 46 (5.5%) | 16 (5.0%) | 30 (5.7%) |  |
| **Mitotic rate** |  |  |  | 0.348 |
| ≤5 | 452 (53.8%) | 164 (51.7%) | 288 (55.1%) |  |
| ＞5 | 388 (46.2%) | 153 (48.3%) | 235 (44.9%) |  |
| Survival months, median (IQR) | 66.0(45.0,94.8) | 64.0(43.0,94.5) | 67.0(46.0,95.0) | 0.277 |

LR: lymph node removed; Others: American Indian, Alaska Native, Asian/Pacifc Islander; IQR: interquartile range
