# Supplementary material for: Influence of lymph node removal on the prognosis of high malignancy potential gastric gastrointestinal stromal tumors: Insights from population-based study
Source: PLoS One. 2024 Dec 5;19(12):e0314504. doi: 10.1371/journal.pone.0314504 (PMC11620419; doi:10.1371/journal.pone.0314504)
Supplement: S3 Table — (DOCX) [file pone.0314504.s003.docx]

**Supplementary Table 3.** Information related to the LR group

| Variables | Total (n=317) |
| --- | --- |
| Regional nodes examined, n (%)  Yes  No  Unknown | 308 (97.2)  8 (2.5)  1 (0.3) |
| Regional nodes positive, n (%)  Yes  No  Unknown | 23 (7.3)  293(92.4)  1 (0.3) |

LR: lymph nodes removed
