# Supplementary material for: Influence of lymph node removal on the prognosis of high malignancy potential gastric gastrointestinal stromal tumors: Insights from population-based study
Source: PLoS One. 2024 Dec 5;19(12):e0314504. doi: 10.1371/journal.pone.0314504 (PMC11620419; doi:10.1371/journal.pone.0314504)
Supplement: Attachment — Submitted filename: Response to Reviewers R1.docx [file pone.0314504.s005.docx]

Dear editor and reviewers of **PLOS ONE**:

Our reference: PONE-D-24-35729

Title: Influence of Lymph Node Removal on the Prognosis of High Malignancy Potential Gastric Gastrointestinal Stromal Tumors: Insights from Population-Based Study

By: Zhenguo Qiao et al

Thank you very much for your letter and for the editors’ and reviewers’ comments concerning our manuscript entitled “Influence of Lymph Node Removal on the Prognosis of High Malignancy Potential Gastric Gastrointestinal Stromal Tumors: Insights from Population-Based Study” (ID: PONE-D-24-35729). These comments are of great reference value to the revision and improvement of our paper and have important guiding significance to our researches. We have studied comments carefully and have made correction. We hope that the revision is acceptable and look forward to hearing from you soon. Revised portion are marked in color in the paper. The main corrections in the paper and the responds to the reviewer’s comments are as flowing:

**Journal Requirements:**

1. When submitting your revision, we need you to address these additional requirements.

Please ensure that your manuscript meets PLOS ONE's style requirements, including those for file naming. The PLOS ONE style templates can be found at 
https://journals.plos.org/plosone/s/file?id=wjVg/PLOSOne_formatting_sample_main_body.pdf and 
https://journals.plos.org/plosone/s/file?id=ba62/PLOSOne_formatting_sample_title_authors_affiliations.pdf

**Response**

Yes, we ensure that our manuscript meets PLOS ONE's style requirements.

2. Thank you for stating in your Funding Statement: 
This work was supported by the Science and Technology Development Program of Suzhou (SKYXD2022041, SKJYD2021184), Suzhou “Science and Education” Youth Science and Technology Project (KJXW2023080), Suzhou City Key Clinical Disease Diagnosis and Treatment Technology Special Project (LCZX202129). There was no additional external funding received for this study.

Please provide an amended statement that declares *all* the funding or sources of support (whether external or internal to your organization) received during this study, as detailed online in our guide for authors at http://journals.plos.org/plosone/s/submit-now.  Please also include the statement “There was no additional external funding received for this study.” in your updated Funding Statement.

Please respond by return e-mail so that we can amend your financial disclosure and competing interests on your behalf.

**Response**

We provide an amended statement:

This work was supported by the Science and Technology Development Program of Suzhou (SKYXD2022041, SKJYD2021184), Suzhou “Science and Education” Youth Science and Technology Project (KJXW2023080), Suzhou City Key Clinical Disease Diagnosis and Treatment Technology Special Project (LCZX202129). All the funding or sources of support received during this study. There was no additional external funding received for this study.

3. Please note that your Data Availability Statement is currently missing the repository name. If your manuscript is accepted for publication, you will be asked to provide these details on a very short timeline. We therefore suggest that you provide this information now, though we will not hold up the peer review process if you are unable.

**Response**

We provide an amended statement:

Publicly available datasets were analyzed in this study. These data can be found in the SEER database: https://seer.cancer.gov/. The datasets supporting the conclusions of this article are included within the article.

4. Please include captions for your Supporting Information files at the end of your manuscript, and update any in-text citations to match accordingly. Please see our Supporting Information guidelines for more information: http://journals.plos.org/plosone/s/supporting-information.

**Response**

We provide captions for our Supporting Information files at the end of our manuscript.

5. Please review your reference list to ensure that it is complete and correct. If you have cited papers that have been retracted, please include the rationale for doing so in the manuscript text, or remove these references and replace them with relevant current references. Any changes to the reference list should be mentioned in the rebuttal letter that accompanies your revised manuscript. If you need to cite a retracted article, indicate the article’s retracted status in the References list and also include a citation and full reference for the retraction notice.

**Response**

We have reviewed our reference list to ensure that it is complete and correct.

**Reviewer #1:** First of all, I must say that I find this work very interesting, pertinent, and relevant because it addresses a topic that has been little studied in GIST, which could have an impact on the treatment and prognosis of patients.
**Comment 1**
There are a few minor grammatical issues (such as incorrect subject-verb agreement, for example in the phrase "HMP-gGISTs patients was gathered"). Some phrases could have greater impact, such as using "our findings suggest that..." instead of "LR may not improve the prognosis of patients with HMP-gGISTs."
**Response 1**

Thank you for your valuable comments on our paper. We appreciate your meticulous review and constructive suggestions for improvement. Regarding the grammatical issues you pointed out, we acknowledge the error in the phrase "HMP-gGISTs patients was gathered" and will correct it to "patients with HMP-gGISTs were gathered" to ensure proper subject-verb agreement. Additionally, we understand the importance of enhancing the impact of our phrases and will revise the sentence "LR may not improve the prognosis of patients with HMP-gGISTs" to "our findings suggest that LR may not improve the prognosis of patients with HMP-gGISTs," as you suggested. Here is the revised paper incorporating your comments: “High malignancy potential gastric gastrointestinal stromal tumors (HMP-gGISTs) generally require surgical resection. However, the necessity of lymph node removal (LR) for patients with such tumors remains unclear. Therefore, we conducted a population-based study to analyze the impact of LR on the long-term prognosis of patients with HMP-gGISTs. Patients with HMP-gGISTs were gathered from the Surveillance, Epidemiology, and End Results (SEER) database. Propensity score matching (PSM) was utilized to address potential selection bias. Overall survival (OS) and cancer-specific survival (CSS) were evaluated using Kaplan-Meier analyses and multivariate Cox proportional hazards models. A total of 840 patients with HMP-gGISTs were included in the study, with 317 undergoing LR and 523 not undergoing LR. The prognosis for OS (P = 0.026) and CSS (P < 0.001) in the LR group was worse compared to the No-LR group. After PSM, 634 patients were matched for comparison. The results showed that the OS (P = 0.028) and CSS (P = 0.006) in the LR group remained poorer than those in the No-LR group. Subgroup analysis further indicated that patients who did not undergo LR had a better prognosis. Our findings suggest that LR may not improve the prognosis of patients with HMP-gGISTs, implying that LR may not be necessary for these patients.”

**Comment 2**
In the demographic characteristics, I would have liked to know the site of the primary tumor and the tumor stage, including lymph node involvement or not, and to see this data for each group of patients with or without LR. These characteristics could also have been used in the PSM, as they have a prognostic impact in GIST patients.
**Response 2**

Thank you for your valuable feedback on our manuscript. We appreciate your suggestion to include the site of the primary tumor and the tumor stage, including lymph node involvement, in the demographic characteristics for each group of patients with or without LR. In response to your comment, we would like to clarify that all the tumors included in our study were primary gastric gastrointestinal stromal tumors (GISTs), as stated in the title and throughout the manuscript. Regarding the tumor stage and lymph node involvement, we acknowledge the importance of these characteristics in prognostic assessments for GIST patients. However, upon carefully reviewing the data from the SEER database after applying our inclusion and exclusion criteria, we found that the data on tumor stage, specifically the TNM staging system, was missing for a significant proportion of patients (74.7%). Given the severity of this data missingness, we decided not to include tumor stage in our analysis to avoid potential bias and ensure the robustness of our findings. We fully recognize the value of incorporating such prognostic factors in future studies. Therefore, in our subsequent research, we plan to collaborate with institutions that have more complete datasets, including detailed information on tumor stage and lymph node involvement. Thank you again for your insightful comments. We are committed to improving our research based on your feedback and look forward to contributing further to the understanding of HMP-gGISTs.

**Comment 3**
I believe the authors' hypotheses regarding the negative impact of lymph node resection surgery are correct, but some limitations or confounding factors could be included, such as: It is possible that patients with greater nodal involvement found during surgery are more likely to undergo lymph node resection, and their prognosis may be affected not only by the surgery itself but also by the stage of the disease. One of the authors' hypotheses is that lymph node resection surgery could affect survival due to a higher risk of surgical complications. The hypothesis is acceptable and plausible; however, it would have been useful to obtain information on surgical complications in both groups to better support the claim they make.
**Response 3**

Thank you for your thoughtful comments on our manuscript. We appreciate your acknowledgment of our hypotheses regarding the negative impact of lymph node resection surgery and your suggestion to consider additional limitations or confounding factors. We fully recognize that patients with greater nodal involvement found during surgery may be more likely to undergo lymph node resection, and their prognosis could indeed be influenced by both the surgery and the stage of the disease. This is an important point that we did not fully address in our original manuscript. In future studies, we will strive to include more detailed information on tumor stage and nodal involvement to better understand their impact on prognosis. Regarding our hypothesis that lymph node resection surgery could affect survival due to a higher risk of surgical complications, we acknowledge the importance of obtaining information on surgical complications in both groups to support our claim. Unfortunately, the SEER database, which was our primary data source, does not provide detailed information on surgical complications. In future research, we plan to collaborate with institutions that have access to more granular data, including surgical complications, to further validate our hypothesis. In the meantime, we have described the lack of information on postoperative complications in the limitation section of the discussion. Thank you again for your valuable feedback. We are committed to improving our research based on your suggestions and look forward to contributing further to the understanding of this important topic.

**Comment 4**
Regarding the study's limitations, I agree with the limitations stated by the authors, but I would perhaps add: The duration of adjuvant treatment (imatinib) is unknown, which could impact survival, as there are studies supporting the use of 1, 2, 3, 5, or even 6 years of adjuvant therapy, and this could influence survival outcomes (I understand that this information is not available in the SEER database). Another limitation is that the primary site of the GIST is unknown, which is also a factor that influences prognosis.
**Response 4**

We appreciate the reviewer's insightful comment and would like to acknowledge an additional limitation of our study. Indeed, the duration of adjuvant treatment (imatinib) is unknown, which could potentially impact survival outcomes. As correctly pointed out, there are studies supporting the use of varying durations of adjuvant therapy, ranging from 1 to 6 years. This information, however, is not available in the SEER database and therefore could not be included in our analysis. We recognize that this is an important factor that could influence the prognosis of patients with HMP-gGISTs and should be considered in future research. We will ensure to highlight this limitation in the revised manuscript to provide a more comprehensive understanding of the potential factors that could affect survival outcomes in this patient population. However, concerning the primary site of the GIST, we would like to clarify that our study specifically focused on gastric gastrointestinal stromal tumors, which by definition have their primary site in the stomach. Therefore, the primary site of the tumors included in our study was indeed known and was consistently the stomach. We apologize for any confusion that may have arisen regarding this point and have clarified it in the revised manuscript to ensure accuracy. Here is the revised paper incorporating your comments: “Our study has several limitations. Firstly, it is a retrospective analysis conducted using the SEER database, which inherently leads to potential data omissions and biases. Nonetheless, we employed MI and PSM techniques to mitigate the effects of missing data and selection bias. Secondly, The SEER database does not contain data regarding postoperative complications, recurrence, margin status, chemotherapy regimen, and genetic mutations of KIT or PDGFRA, all of which may have an impact on the long-term prognosis of patients. Thirdly, information regarding tumor rupture status in GIST surgery patients is absent from the SEER database, and utilizing our own clinical data for subsequent analysis might allow for improved stratification of patients according to their malignant potential. Lastly, the duration of adjuvant treatment (imatinib) is unknown, which could impact survival, as there are studies supporting the use of 1, 2, 3, 5, or even 6 years of adjuvant therapy, and this could influence survival outcomes.”

**Comment 5**
In conclusion, I believe that, despite its limitations, this is a pertinent and interesting study that merits publication. I would suggest reviewing the minor grammatical errors and encourage the authors to expand on the hypotheses regarding the results obtained and the study’s limitations.

**Response 5**

Thank you very much for your positive feedback and constructive comments on our manuscript. We are glad to hear that you find our study pertinent and interesting, and we appreciate your encouragement for publication. We have carefully reviewed the manuscript for minor grammatical errors and will make the necessary corrections to ensure the clarity and readability of the text. Regarding your suggestion to expand on the hypotheses regarding the results obtained and the study’s limitations, we would like to inform you that we have already addressed this in the revised manuscript. Additionally, we have also expanded the limitation section to include a more comprehensive discussion of the potential factors that could affect our results, such as the lack of information on adjuvant treatment duration, as you have previously pointed out. We believe that these additions will enhance the quality and completeness of our study. Once again, we would like to express our gratitude for your valuable comments and suggestions. We are confident that the revisions we have made will further improve the manuscript and make it more suitable for publication. Thank you for your time and consideration.

**Reviewer #2:** This population-based study addresses a critical gap in understanding the role of lymph node removal in patients with HMP-gGISTs. While surgical resection is the mainstay treatment, the necessity of LR has long remained unclear. The results highlight that patients who underwent LR had worse overall survival (OS) and cancer-specific survival (CSS) compared to those who did not undergo LR, both before and after PSM. While this study suggests that LR may not improve outcomes, further prospective studies and clinical trials could help validate these findings and refine guidelines for managing HMP-gGISTs. Additionally, understanding the biological reasons behind the poorer outcomes in the LR group could open new avenues for research.

**Response**

Thank you very much for your thoughtful and comprehensive review of our manuscript. We are grateful for your acknowledgment of the critical gap our study addresses in understanding the role of LR in patients with HMP-gGISTs. We appreciate your summary of our key findings, which highlight the worse OS and CSS in patients who underwent LR compared to those who did not, both before and after PSM. Your suggestion for further prospective studies and clinical trials to validate our findings and refine guidelines for managing HMP-gGISTs is invaluable. We completely agree that such studies are necessary to confirm our results and contribute to the evidence-based management of these patients. Moreover, we are intrigued by your suggestion to explore the biological reasons behind the poorer outcomes in the LR group. This is indeed a fascinating area for future research, and we will consider incorporating this perspective into our ongoing and future studies. Thank you once again for your insightful comments and constructive suggestions. We are encouraged by your positive feedback and look forward to the opportunity to further refine our research based on your guidance.

Once again, thank you very much for your comments and suggestions.

Yours sincerely,

Yang L
2024-11-10
