# Supplementary material for: Influence of lymph node removal on the prognosis of high malignancy potential gastric gastrointestinal stromal tumors: Insights from population-based study
Source: PLoS One. 2024 Dec 5;19(12):e0314504. doi: 10.1371/journal.pone.0314504 (PMC11620419; doi:10.1371/journal.pone.0314504)
Supplement: Peer review history

# Peer review: Decision Letter 0

**DOI:** 10.1371/journal.pone.0314504.r001

**Authors:** Paolo Aurello

8 Nov 2024

PONE-D-24-35729Influence of Lymph Node Removal on the Prognosis of High Malignancy Potential Gastric Gastrointestinal Stromal Tumors: Insights from Population-Based StudyPLOS ONE

Dear Dr. yang,

Thank you for submitting your manuscript to PLOS ONE. After careful consideration, we feel that it has merit but does not fully meet PLOS ONE’s publication criteria as it currently stands. Therefore, we invite you to submit a revised version of the manuscript that addresses the points raised during the review process.

Please submit your revised manuscript by Dec 22 2024 11:59PM. If you will need more time than this to complete your revisions, please reply to this message or contact the journal office at plosone@plos.org. When you're ready to submit your revision, log on to https://www.editorialmanager.com/pone/ and select the 'Submissions Needing Revision' folder to locate your manuscript file.

Please include the following items when submitting your revised manuscript:

- A rebuttal letter that responds to each point raised by the academic editor and reviewer(s). You should upload this letter as a separate file labeled 'Response to Reviewers'.
- A marked-up copy of your manuscript that highlights changes made to the original version. You should upload this as a separate file labeled 'Revised Manuscript with Track Changes'.
- An unmarked version of your revised paper without tracked changes. You should upload this as a separate file labeled 'Manuscript'.

If you would like to make changes to your financial disclosure, please include your updated statement in your cover letter. Guidelines for resubmitting your figure files are available below the reviewer comments at the end of this letter.

If applicable, we recommend that you deposit your laboratory protocols in protocols.io to enhance the reproducibility of your results. Protocols.io assigns your protocol its own identifier (DOI) so that it can be cited independently in the future. For instructions see: https://journals.plos.org/plosone/s/submission-guidelines#loc-laboratory-protocols. Additionally, PLOS ONE offers an option for publishing peer-reviewed Lab Protocol articles, which describe protocols hosted on protocols.io. Read more information on sharing protocols at https://plos.org/protocols?utm_medium=editorial-email&utm_source=authorletters&utm_campaign=protocols.

We look forward to receiving your revised manuscript.

Kind regards,

Marianne Clemence, Staff Editor, on behalf of

Paolo Aurello

Academic Editor

PLOS ONE

**Journal Requirements:**

1. When submitting your revision, we need you to address these additional requirements.

Please ensure that your manuscript meets PLOS ONE's style requirements, including those for file naming. The PLOS ONE style templates can be found at 

https://journals.plos.org/plosone/s/file?id=wjVg/PLOSOne_formatting_sample_main_body.pdf and 

https://journals.plos.org/plosone/s/file?id=ba62/PLOSOne_formatting_sample_title_authors_affiliations.pdf

2. Thank you for stating in your Funding Statement: 

This work was supported by the Science and Technology Development Program of Suzhou (SKYXD2022041, SKJYD2021184), Suzhou “Science and Education” Youth Science and Technology Project (KJXW2023080), Suzhou City Key Clinical Disease Diagnosis and Treatment Technology Special Project (LCZX202129). There was no additional external funding received for this study.

Please provide an amended statement that declares *all* the funding or sources of support (whether external or internal to your organization) received during this study, as detailed online in our guide for authors at http://journals.plos.org/plosone/s/submit-now.  Please also include the statement “There was no additional external funding received for this study.” in your updated Funding Statement. 

Please respond by return e-mail so that we can amend your financial disclosure and competing interests on your behalf.

3. Please note that your Data Availability Statement is currently missing the repository name. If your manuscript is accepted for publication, you will be asked to provide these details on a very short timeline. We therefore suggest that you provide this information now, though we will not hold up the peer review process if you are unable.

4. Please include captions for your Supporting Information files at the end of your manuscript, and update any in-text citations to match accordingly. Please see our Supporting Information guidelines for more information: http://journals.plos.org/plosone/s/supporting-information. 

5. Please review your reference list to ensure that it is complete and correct. If you have cited papers that have been retracted, please include the rationale for doing so in the manuscript text, or remove these references and replace them with relevant current references. Any changes to the reference list should be mentioned in the rebuttal letter that accompanies your revised manuscript. If you need to cite a retracted article, indicate the article’s retracted status in the References list and also include a citation and full reference for the retraction notice.

[Note: HTML markup is below. Please do not edit.]

Reviewers' comments:

Reviewer's Responses to Questions

**Comments to the Author**

1. Is the manuscript technically sound, and do the data support the conclusions?

The manuscript must describe a technically sound piece of scientific research with data that supports the conclusions. Experiments must have been conducted rigorously, with appropriate controls, replication, and sample sizes. The conclusions must be drawn appropriately based on the data presented. 

Reviewer #1: Yes

Reviewer #2: Yes

**********

2. Has the statistical analysis been performed appropriately and rigorously? 

Reviewer #1: Yes

Reviewer #2: Yes

**********

3. Have the authors made all data underlying the findings in their manuscript fully available?

The PLOS Data policy requires authors to make all data underlying the findings described in their manuscript fully available without restriction, with rare exception (please refer to the Data Availability Statement in the manuscript PDF file). The data should be provided as part of the manuscript or its supporting information, or deposited to a public repository. For example, in addition to summary statistics, the data points behind means, medians and variance measures should be available. If there are restrictions on publicly sharing data—e.g. participant privacy or use of data from a third party—those must be specified.

Reviewer #1: Yes

Reviewer #2: Yes

**********

4. Is the manuscript presented in an intelligible fashion and written in standard English?

PLOS ONE does not copyedit accepted manuscripts, so the language in submitted articles must be clear, correct, and unambiguous. Any typographical or grammatical errors should be corrected at revision, so please note any specific errors here.

Reviewer #1: Yes

Reviewer #2: Yes

**********

5. Review Comments to the Author

Please use the space provided to explain your answers to the questions above. You may also include additional comments for the author, including concerns about dual publication, research ethics, or publication ethics. (Please upload your review as an attachment if it exceeds 20,000 characters)

**Reviewer #1:** First of all, I must say that I find this work very interesting, pertinent, and relevant because it addresses a topic that has been little studied in GIST, which could have an impact on the treatment and prognosis of patients.

There are a few minor grammatical issues (such as incorrect subject-verb agreement, for example in the phrase "HMP-gGISTs patients was gathered"). Some phrases could have greater impact, such as using "our findings suggest that..." instead of "LR may not improve the prognosis of patients with HMP-gGISTs."

In the demographic characteristics, I would have liked to know the site of the primary tumor and the tumor stage, including lymph node involvement or not, and to see this data for each group of patients with or without LR. These characteristics could also have been used in the PSM, as they have a prognostic impact in GIST patients.

I believe the authors' hypotheses regarding the negative impact of lymph node resection surgery are correct, but some limitations or confounding factors could be included, such as: It is possible that patients with greater nodal involvement found during surgery are more likely to undergo lymph node resection, and their prognosis may be affected not only by the surgery itself but also by the stage of the disease. One of the authors' hypotheses is that lymph node resection surgery could affect survival due to a higher risk of surgical complications. The hypothesis is acceptable and plausible; however, it would have been useful to obtain information on surgical complications in both groups to better support the claim they make.

Regarding the study's limitations, I agree with the limitations stated by the authors, but I would perhaps add: The duration of adjuvant treatment (imatinib) is unknown, which could impact survival, as there are studies supporting the use of 1, 2, 3, 5, or even 6 years of adjuvant therapy, and this could influence survival outcomes (I understand that this information is not available in the SEER database). Another limitation is that the primary site of the GIST is unknown, which is also a factor that influences prognosis.

In conclusion, I believe that, despite its limitations, this is a pertinent and interesting study that merits publication. I would suggest reviewing the minor grammatical errors and encourage the authors to expand on the hypotheses regarding the results obtained and the study’s limitations.

**Reviewer #2: **This population-based study addresses a critical gap in understanding the role of lymph node removal in patients with HMP-gGISTs. While surgical resection is the mainstay treatment, the necessity of LR has long remained unclear. he results highlight that patients who underwent LR had worse overall survival (OS) and cancer-specific survival (CSS) compared to those who did not undergo LR, both before and after PSM. While this study suggests that LR may not improve outcomes, further prospective studies and clinical trials could help validate these findings and refine guidelines for managing HMP-gGISTs. Additionally, understanding the biological reasons behind the poorer outcomes in the LR group could open new avenues for research.

**********

6. PLOS authors have the option to publish the peer review history of their article (what does this mean?). If published, this will include your full peer review and any attached files.

If you choose “no”, your identity will remain anonymous but your review may still be made public.

**Do you want your identity to be public for this peer review?** For information about this choice, including consent withdrawal, please see our Privacy Policy.

Reviewer #1: No

Reviewer #2: **Yes: **Suresh VS Attili

**********

[NOTE: If reviewer comments were submitted as an attachment file, they will be attached to this email and accessible via the submission site. Please log into your account, locate the manuscript record, and check for the action link "View Attachments". If this link does not appear, there are no attachment files.]

While revising your submission, please upload your figure files to the Preflight Analysis and Conversion Engine (PACE) digital diagnostic tool, https://pacev2.apexcovantage.com/. PACE helps ensure that figures meet PLOS requirements. To use PACE, you must first register as a user. Registration is free. Then, login and navigate to the UPLOAD tab, where you will find detailed instructions on how to use the tool. If you encounter any issues or have any questions when using PACE, please email PLOS at figures@plos.org. Please note that Supporting Information files do not need this step.

# Author comment: Author response to Decision Letter 0

**DOI:** 10.1371/journal.pone.0314504.r002

9 Nov 2024

Dear editor and reviewers of PLOS ONE:

Our reference: PONE-D-24-35729

Title: Influence of Lymph Node Removal on the Prognosis of High Malignancy Potential Gastric Gastrointestinal Stromal Tumors: Insights from Population-Based Study

By: Zhenguo Qiao et al

Thank you very much for your letter and for the editors’ and reviewers’ comments concerning our manuscript entitled “Influence of Lymph Node Removal on the Prognosis of High Malignancy Potential Gastric Gastrointestinal Stromal Tumors: Insights from Population-Based Study” (ID: PONE-D-24-35729). These comments are of great reference value to the revision and improvement of our paper and have important guiding significance to our researches. We have studied comments carefully and have made correction. We hope that the revision is acceptable and look forward to hearing from you soon. Revised portion are marked in color in the paper. The main corrections in the paper and the responds to the reviewer’s comments are as flowing:

Journal Requirements:

1. When submitting your revision, we need you to address these additional requirements.

Please ensure that your manuscript meets PLOS ONE's style requirements, including those for file naming. The PLOS ONE style templates can be found at 

https://journals.plos.org/plosone/s/file?id=wjVg/PLOSOne_formatting_sample_main_body.pdf and 

https://journals.plos.org/plosone/s/file?id=ba62/PLOSOne_formatting_sample_title_authors_affiliations.pdf

Response 

Yes, we ensure that our manuscript meets PLOS ONE's style requirements.

2. Thank you for stating in your Funding Statement: 

This work was supported by the Science and Technology Development Program of Suzhou (SKYXD2022041, SKJYD2021184), Suzhou “Science and Education” Youth Science and Technology Project (KJXW2023080), Suzhou City Key Clinical Disease Diagnosis and Treatment Technology Special Project (LCZX202129). There was no additional external funding received for this study.

Please provide an amended statement that declares *all* the funding or sources of support (whether external or internal to your organization) received during this study, as detailed online in our guide for authors at http://journals.plos.org/plosone/s/submit-now.  Please also include the statement “There was no additional external funding received for this study.” in your updated Funding Statement. 

Please respond by return e-mail so that we can amend your financial disclosure and competing interests on your behalf.

Response 

We provide an amended statement: 

This work was supported by the Science and Technology Development Program of Suzhou (SKYXD2022041, SKJYD2021184), Suzhou “Science and Education” Youth Science and Technology Project (KJXW2023080), Suzhou City Key Clinical Disease Diagnosis and Treatment Technology Special Project (LCZX202129). All the funding or sources of support received during this study. There was no additional external funding received for this study.

3.Please note that your Data Availability Statement is currently missing the repository name. If your manuscript is accepted for publication, you will be asked to provide these details on a very short timeline. We therefore suggest that you provide this information now, though we will not hold up the peer review process if you are unable.

Response 

We provide an amended statement: 

Publicly available datasets were analyzed in this study. These data can be found in the SEER database: https://seer.cancer.gov/. The datasets supporting the conclusions of this article are included within the article.

4.Please include captions for your Supporting Information files at the end of your manuscript, and update any in-text citations to match accordingly. Please see our Supporting Information guidelines for more information: http://journals.plos.org/plosone/s/supporting-information. 

Response 

We provide captions for our Supporting Information files at the end of our manuscript.

5.Please review your reference list to ensure that it is complete and correct. If you have cited papers that have been retracted, please include the rationale for doing so in the manuscript text, or remove these references and replace them with relevant current references. Any changes to the reference list should be mentioned in the rebuttal letter that accompanies your revised manuscript. If you need to cite a retracted article, indicate the article’s retracted status in the References list and also include a citation and full reference for the retraction notice.

Response 

We have reviewed our reference list to ensure that it is complete and correct.

Reviewer #1: First of all, I must say that I find this work very interesting, pertinent, and relevant because it addresses a topic that has been little studied in GIST, which could have an impact on the treatment and prognosis of patients.

Comment 1

There are a few minor grammatical issues (such as incorrect subject-verb agreement, for example in the phrase "HMP-gGISTs patients was gathered"). Some phrases could have greater impact, such as using "our findings suggest that..." instead of "LR may not improve the prognosis of patients with HMP-gGISTs."

Response 1

Thank you for your valuable comments on our paper. We appreciate your meticulous review and constructive suggestions for improvement. Regarding the grammatical issues you pointed out, we acknowledge the error in the phrase "HMP-gGISTs patients was gathered" and will correct it to "patients with HMP-gGISTs were gathered" to ensure proper subject-verb agreement. Additionally, we understand the importance of enhancing the impact of our phrases and will revise the sentence "LR may not improve the prognosis of patients with HMP-gGISTs" to "our findings suggest that LR may not improve the prognosis of patients with HMP-gGISTs," as you suggested. Here is the revised paper incorporating your comments: “High malignancy potential gastric gastrointestinal stromal tumors (HMP-gGISTs) generally require surgical resection. However, the necessity of lymph node removal (LR) for patients with such tumors remains unclear. Therefore, we conducted a population-based study to analyze the impact of LR on the long-term prognosis of patients with HMP-gGISTs. Patients with HMP-gGISTs were gathered from the Surveillance, Epidemiology, and End Results (SEER) database. Propensity score matching (PSM) was utilized to address potential selection bias. Overall survival (OS) and cancer-specific survival (CSS) were evaluated using Kaplan-Meier analyses and multivariate Cox proportional hazards models. A total of 840 patients with HMP-gGISTs were included in the study, with 317 undergoing LR and 523 not undergoing LR. The prognosis for OS (P = 0.026) and CSS (P < 0.001) in the LR group was worse compared to the No-LR group. After PSM, 634 patients were matched for comparison. The results showed that the OS (P = 0.028) and CSS (P = 0.006) in the LR group remained poorer than those in the No-LR group. Subgroup analysis further indicated that patients who did not undergo LR had a better prognosis. Our findings suggest that LR may not improve the prognosis of patients with HMP-gGISTs, implying that LR may not be necessary for these patients.”

Comment 2

In the demographic characteristics, I would have liked to know the site of the primary tumor and the tumor stage, including lymph node involvement or not, and to see this data for each group of patients with or without LR. These characteristics could also have been used in the PSM, as they have a prognostic impact in GIST patients.

Response 2

Thank you for your valuable feedback on our manuscript. We appreciate your suggestion to include the site of the primary tumor and the tumor stage, including lymph node involvement, in the demographic characteristics for each group of patients with or without LR. In response to your comment, we would like to clarify that all the tumors included in our study were primary gastric gastrointestinal stromal tumors (GISTs), as stated in the title and throughout the manuscript. Regarding the tumor stage and lymph node involvement, we acknowledge the importance of these characteristics in prognostic assessments for GIST patients. However, upon carefully reviewing the data from the SEER database after applying our inclusion and exclusion criteria, we found that the data on tumor stage, specifically the TNM staging system, was missing for a significant proportion of patients (74.7%). Given the severity of this data missingness, we decided not to include tumor stage in our analysis to avoid potential bias and ensure the robustness of our findings. We fully recognize the value of incorporating such prognostic factors in future studies. Therefore, in our subsequent research, we plan to collaborate with institutions that have more complete datasets, including detailed information on tumor stage and lymph node involvement. Thank you again for your insightful comments. We are committed to improving our research based on your feedback and look forward to contributing further to the understanding of HMP-gGISTs.

Comment 3

I believe the authors' hypotheses regarding the negative impact of lymph node resection surgery are correct, but some limitations or confounding factors could be included, such as: It is possible that patients with greater nodal involvement found during surgery are more likely to undergo lymph node resection, and their prognosis may be affected not only by the surgery itself but also by the stage of the disease. One of the authors' hypotheses is that lymph node resection surgery could affect survival due to a higher risk of surgical complications. The hypothesis is acceptable and plausible; however, it would have been useful to obtain information on surgical complications in both groups to better support the claim they make.

Response 3

Thank you for your thoughtful comments on our manuscript. We appreciate your acknowledgment of our hypotheses regarding the negative impact of lymph node resection surgery and your suggestion to consider additional limitations or confounding factors. We fully recognize that patients with greater nodal involvement found during surgery may be more likely to undergo lymph node resection, and their prognosis could indeed be influenced by both the surgery and the stage of the disease. This is an important point that we did not fully address in our original manuscript. In future studies, we will strive to include more detailed information on tumor stage and nodal involvement to better understand their impact on prognosis. Regarding our hypothesis that lymph node resection surgery could affect survival due to a higher risk of surgical complications, we acknowledge the importance of obtaining information on surgical complications in both groups to support our claim. Unfortunately, the SEER database, which was our primary data source, does not provide detailed information on surgical complications. In future research, we plan to collaborate with institutions that have access to more granular data, including surgical complications, to further validate our hypothesis. In the meantime, we have described the lack of information on postoperative complications in the limitation section of the discussion. Thank you again for your valuable feedback. We are committed to improving our research based on your suggestions and look forward to contributing further to the understanding of this important topic.

Comment 4

Regarding the study's limitations, I agree with the limitations stated by the authors, but I would perhaps add: The duration of adjuvant treatment (imatinib) is unknown, which could impact survival, as there are studies supporting the use of 1, 2, 3, 5, or even 6 years of adjuvant therapy, and this could influence survival outcomes (I understand that this information is not available in the SEER database). Another limitation is that the primary site of the GIST is unknown, which is also a factor that influences prognosis.

Response 4

We appreciate the reviewer's insightful comment and would like to acknowledge an additional limitation of our study. Indeed, the duration of adjuvant treatment (imatinib) is unknown, which could potentially impact survival outcomes. As correctly pointed out, there are studies supporting the use of varying durations of adjuvant therapy, ranging from 1 to 6 years. This information, however, is not available in the SEER database and therefore could not be included in our analysis. We recognize that this is an important factor that could influence the prognosis of patients with HMP-gGISTs and should be considered in future research. We will ensure to highlight this limitation in the revised manuscript to provide a more comprehensive understanding of the potential factors that could affect survival outcomes in this patient population. However, concerning the primary site of the GIST, we would like to clarify that our study specifically focused on gastric gastrointestinal stromal tumors, which by definition have their primary site in the stomach. Therefore, the primary site of the tumors included in our study was indeed known and was consistently the stomach. We apologize for any confusion that may have arisen regarding this point and have clarified it in the revised manuscript to ensure accuracy. Here is the revised paper incorporating your comments: “Our study has several limitations. Firstly, it is a retrospective analysis conducted using the SEER database, which inherently leads to potential data omissions and biases. Nonetheless, we employed MI and PSM techniques to mitigate the effects of missing data and selection bias. Secondly, The SEER database does not contain data regarding postoperative complications, recurrence, margin status, chemotherapy regimen, and genetic mutations of KIT or PDGFRA, all of which may have an impact on the long-term prognosis of patients. Thirdly, information regarding tumor rupture status in GIST surgery patients is absent from the SEER database, and utilizing our own clinical data for subsequent analysis might allow for improved stratification of patients according to their malignant potential. Lastly, the duration of adjuvant treatment (imatinib) is unknown, which could impact survival, as there are studies supporting the use of 1, 2, 3, 5, or even 6 years of adjuvant therapy, and this could influence survival outcomes.”

Comment 5

In conclusion, I believe that, despite its limitations, this is a pertinent and interesting study that merits publication. I would suggest reviewing the minor grammatical errors and encourage the authors to expand on the hypotheses regarding the results obtained and the study’s limitations.

Response 5

Thank you very much for your positive feedback and constructive comments on our manuscript. We are glad to hear that you find our study pertinent and interesting, and we appreciate your encouragement for publication. We have carefully reviewed the manuscript for minor grammatical errors and will make the necessary corrections to ensure the clarity and readability of the text. Regarding your suggestion to expand on the hypotheses regarding the results obtained and the study’s limitations, we would like to inform you that we have already addressed this in the revised manuscript. Additionally, we have also expanded the limitation section to include a more comprehensive discussion of the potential factors that could affect our results, such as the lack of information on adjuvant treatment duration, as you have previously pointed out. We believe that these additions will enhance the quality and completeness of our study. Once again, we would like to express our gratitude for your valuable comments and suggestions. We are confident that the revisions we have made will further improve the manuscript and make it more suitable for publication. Thank you for your time and consideration.

Reviewer #2: This population-based study addresses a critical gap in understanding the role of lymph node removal in patients with HMP-gGISTs. While surgical resection is the mainstay treatment, the necessity of LR has long remained unclear. The results highlight that patients who underwent LR had worse overall survival (OS) and cancer-specific survival (CSS) compared to those who did not undergo LR, both before and after PSM. While this st

Attachment Submitted filename: Response to Reviewers R1.docx

# Editor report: Decision Letter 1

**DOI:** 10.1371/journal.pone.0314504.r003

**Authors:** Paolo Aurello

12 Nov 2024

Influence of Lymph Node Removal on the Prognosis of High Malignancy Potential Gastric Gastrointestinal Stromal Tumors: Insights from Population-Based Study

PONE-D-24-35729R1

Dear Dr. lingxia yang,

We’re pleased to inform you that your manuscript has been judged scientifically suitable for publication and will be formally accepted for publication once it meets all outstanding technical requirements.

Within one week, you’ll receive an e-mail detailing the required amendments. When these have been addressed, you’ll receive a formal acceptance letter and your manuscript will be scheduled for publication.

An invoice will be generated when your article is formally accepted. Please note, if your institution has a publishing partnership with PLOS and your article meets the relevant criteria, all or part of your publication costs will be covered. Please make sure your user information is up-to-date by logging into Editorial Manager at Editorial Manager® and clicking the ‘Update My Information' link at the top of the page. If you have any questions relating to publication charges, please contact our Author Billing department directly at authorbilling@plos.org.

If your institution or institutions have a press office, please notify them about your upcoming paper to help maximize its impact. If they’ll be preparing press materials, please inform our press team as soon as possible -- no later than 48 hours after receiving the formal acceptance. Your manuscript will remain under strict press embargo until 2 pm Eastern Time on the date of publication. For more information, please contact onepress@plos.org.

Kind regards,

Paolo Aurello

Academic Editor

PLOS ONE

# Editor report: Acceptance letter

**DOI:** 10.1371/journal.pone.0314504.r004

**Authors:** Paolo Aurello

22 Nov 2024

PONE-D-24-35729R1 

PLOS ONE

Dear Dr. yang, 

I'm pleased to inform you that your manuscript has been deemed suitable for publication in PLOS ONE. Congratulations! Your manuscript is now being handed over to our production team.

At this stage, our production department will prepare your paper for publication. This includes ensuring the following:

* All references, tables, and figures are properly cited

* All relevant supporting information is included in the manuscript submission,

* There are no issues that prevent the paper from being properly typeset

If revisions are needed, the production department will contact you directly to resolve them. If no revisions are needed, you will receive an email when the publication date has been set. At this time, we do not offer pre-publication proofs to authors during production of the accepted work. Please keep in mind that we are working through a large volume of accepted articles, so please give us a few weeks to review your paper and let you know the next and final steps. 

Lastly, if your institution or institutions have a press office, please let them know about your upcoming paper now to help maximize its impact. If they'll be preparing press materials, please inform our press team within the next 48 hours. Your manuscript will remain under strict press embargo until 2 pm Eastern Time on the date of publication. For more information, please contact onepress@plos.org.

If we can help with anything else, please email us at customercare@plos.org.

Thank you for submitting your work to PLOS ONE and supporting open access. 

Kind regards, 

PLOS ONE Editorial Office Staff

on behalf of

Dr. Paolo Aurello 

Academic Editor

PLOS ONE
